# Severe asthma in horses is associated with increased airway innervation

**DOI:** 10.1111/jvim.16941

**Published:** 2023-12-06

**Authors:** Laurence Leduc, Mathilde Leclère, Laurie Girardot Gauthier, Olivier Marcil, Jean‐Pierre Lavoie

**Affiliations:** ^1^ Department of Clinical Sciences, Faculté de Médecine Vétérinaire Université de Montréal Saint‐Hyacinthe Quebec Canada; ^2^ Collège André Grasset Montréal Quebec Canada

**Keywords:** heaves, horses, immunohistochemistry, nerves, remodeling

## Abstract

**Background:**

Altered innervation structure and function contribute to airway hyperresponsiveness in human asthma, yet the role of innervation in airflow limitation in asthma in horses remains unknown.

**Hypothesis:**

To characterize peribronchial innervation in horses with asthma. We hypothesized that airway innervation increases in horses with asthma compared with controls.

**Animals:**

Formalin‐fixed lung samples from 8 horses with severe asthma and 8 healthy horses from the Equine Respiratory Tissue Biobank. Ante‐mortem lung function was recorded.

**Methods:**

Blinded case‐control study. Immunohistochemistry was performed using rabbit anti‐s100 antibody as a neuronal marker for myelinating and non‐myelinating Schwann cells. The number and cumulative area of nerves in the peribronchial region and associated with airway smooth muscle were recorded using histomorphometry and corrected for airway size.

**Results:**

Both the number (median [IQR]: 1.87 × 10^−5^ nerves/μm^2^ [1.28 × 10^−5^]) and the cumulative nerve area (CNA; 1.03 × 10^−3^ CNA/μm^2^ [1.57 × 10^−3^]) were higher in the peribronchial region of horses with asthma compared with controls (5.17 × 10^−6^ nerves/μm^2^ [3.76 × 10^−6^], 4.14 × 10^−4^ CNA/μm^2^ [2.54 × 10^−4^], Mann‐Whitney, *P* = .01). The number of nerves within or lining airway smooth muscle was significantly higher in horses with asthma (4.47 × 10^−6^ nerves/μm^2^ [5.75 × 10^−6^]) compared with controls (2.26 × 10^−6^ nerves/μm^2^ [1.16 × 10^−6^], Mann‐Whitney, *P* = .03).

**Conclusions and Clinical Importance:**

Asthma in horses is associated with greater airway innervation, possibly contributing to airway smooth muscle remodeling and exacerbating severity of the disease.

**Abbreviations**
AHR: airway hyperresponsiveness
ASM: airway smooth muscle area
BALF: bronchoalveolar lavage fluid
CNA: cumulative nerve area
CNA‐ASM: smooth muscle‐associated cumulative nerve area
*E*_*L*_
: lung elastance
ERTB: equine respiratory tissue biobank
ID: internal diameter
IP: internal perimeter
IQR: interquartile range
LA: lumen area
MT: morphometric tracing
NBN: number of peribronchial nerves
NBN‐ASM: number of smooth muscle‐associated nerves
NVN: number of perivascular nerves
PC: point counting analysis
*R*_*L*_
: lung resistance
VSM: vascular smooth muscle area

## 1 INTRODUCTION

Severe asthma is a chronic disease affecting approximately 15% of adult horses.^1^ Asthma reduces the quality of life and athletic performance of horses and results in premature retirement and euthanasia.^2^ Airflow obstruction in horses during exacerbations of asthma is largely caused by bronchoconstriction and airway hyperresponsiveness (AHR) in response to inhalation of environmental antigens, as 60% to 70% improvement in lung function is typically observed after the administration of bronchodilators.^3^, ^4^ There is also evidence of mild but persistent airflow limitation that bronchodilators can revert when horses are in clinical remission and under low antigenic stimulation.^5^ Furthermore, coughing, frequently observed in horses with asthma, is a neural reflex.^6^

The presence of AHR in the absence of inflammation highlights how other mechanisms, such as nerve activity, could contribute to the development and persistence of AHR.^7^, ^8^, ^9^ In humans and mice, the sensitivity to inhaled antigens and peribronchial smooth muscle contraction is mostly controlled by afferent sensory nerves, which include myelinated A fibers and unmyelinated C fibers, and efferent myelinated and unmyelinated parasympathetic airway nerves.^10^, ^11^ Impaired airway innervation could contribute to AHR, bronchoconstriction, and airway remodeling as acetylcholine released by nerve endings increases airway smooth muscle mass in mice, and vagotomy prevents AHR and airway inflammation in dogs.^12^, ^13^, ^14^ Altered innervation might not be limited to bronchi as increased length and branching of immunoreactive substance P nerves are observed in vessels and glands of lung tissues in humans with asthma.^15^

Afferent and efferent nerves of the respiratory tract travel via the vagus nerve and regulate not only airway smooth muscle tone and breathing pattern, but can also affect vascular tone, mucus secretion and inflammation via the muscarinic receptors on many cell types.^10^, ^16^, ^17^ Furthermore, innervation may be involved in the airway and vascular smooth muscle remodeling occurring in horses with severe asthma.^18^, ^19^, ^20^ Although there are functional alterations of airway innervation in horses with severe asthma,^21^, ^22^ histological quantification of peribronchial nerves has not yet been investigated, to the knowledge of the authors.

This study aimed to characterize peribronchial innervation in horses with asthma using immunohistochemistry and histomorphometry. We hypothesized that peribronchial innervation (number of nerves and cumulative nerve surface area) would increase in horses with severe asthma compared with controls. Considering that the main effector of peribronchial nerves is the smooth muscle surrounding bronchi and bronchioles, we also hypothesized that smooth muscle‐associated innervation would be increased. Finally, we measured the vascular wall innervation of adjacent pulmonary arteries to investigate whether the increase in vascular innervation was specific to the bronchial wall.

## 2 MATERIALS AND METHODS

### 2.1 Study design

Blinded case‐control study.

### 2.2 Horses

The study was performed using peripheral samples collected post‐mortem from the caudodorsal lungs. Eight horses previously diagnosed with severe asthma (5 in exacerbation and 3 in remission at the time of euthanasia) and 8 healthy horses from the Equine Respiratory Tissue Biobank (ERTB) were included. Ante‐mortem pulmonary function testing, bronchoalveolar lavage fluid (BALF) cytology, housing characteristics, and diet were available for review for each horse. To be included in the ERTB, horses with severe asthma had a documented history of labored breathing at rest and airflow obstruction with pulmonary resistance (*R*_*L*_) > 1 cm H_2_O/L/s, pulmonary elastance (*E*_*L*_) > 1 cm H_2_O/L, and neutrophilic airway inflammation (BALF neutrophils >15%) when exposed to hay. In addition to labored breathing, at least 2 of these 3 criteria were met at the time of euthanasia for horses in exacerbation, which was induced by exposure to dusty hay in a research barn. The duration of exacerbation before euthanasia varied between horses (approximately 2‐6 weeks). Horses in remission had met these criteria in the past but had no abnormal respiratory signs at the time of euthanasia and had at least 2 of the following: *R*_*L*_ ≤ 1.0 cm H_2_O/L/s; *E*_*L*_ ≤ 1.0 cm H_2_O/L; BALF neutrophils ≤10%. These horses had been in an antigen‐poor environment (pasture or hay alternatives and shavings) for 1.5 to 12 months before tissue sampling. Control horses had no history of asthma, no current respiratory signs, and had normal pulmonary function and BALF cytology (*R*_*L*_ ≤ 1 cm H_2_O/L/s, *E*_*L*_ ≤ 1 cm H_2_O/L; BALF neutrophils ≤10%). The horses' BALF neutrophil percentages and pulmonary function testing can be found in Figures S1 and S2. Horses entered the ERTB under the animal use and care protocols associated with the projects they were enrolled in before euthanasia (Rech‐1578).

### 2.3 Immunohistochemistry

**FIGURE 1:**
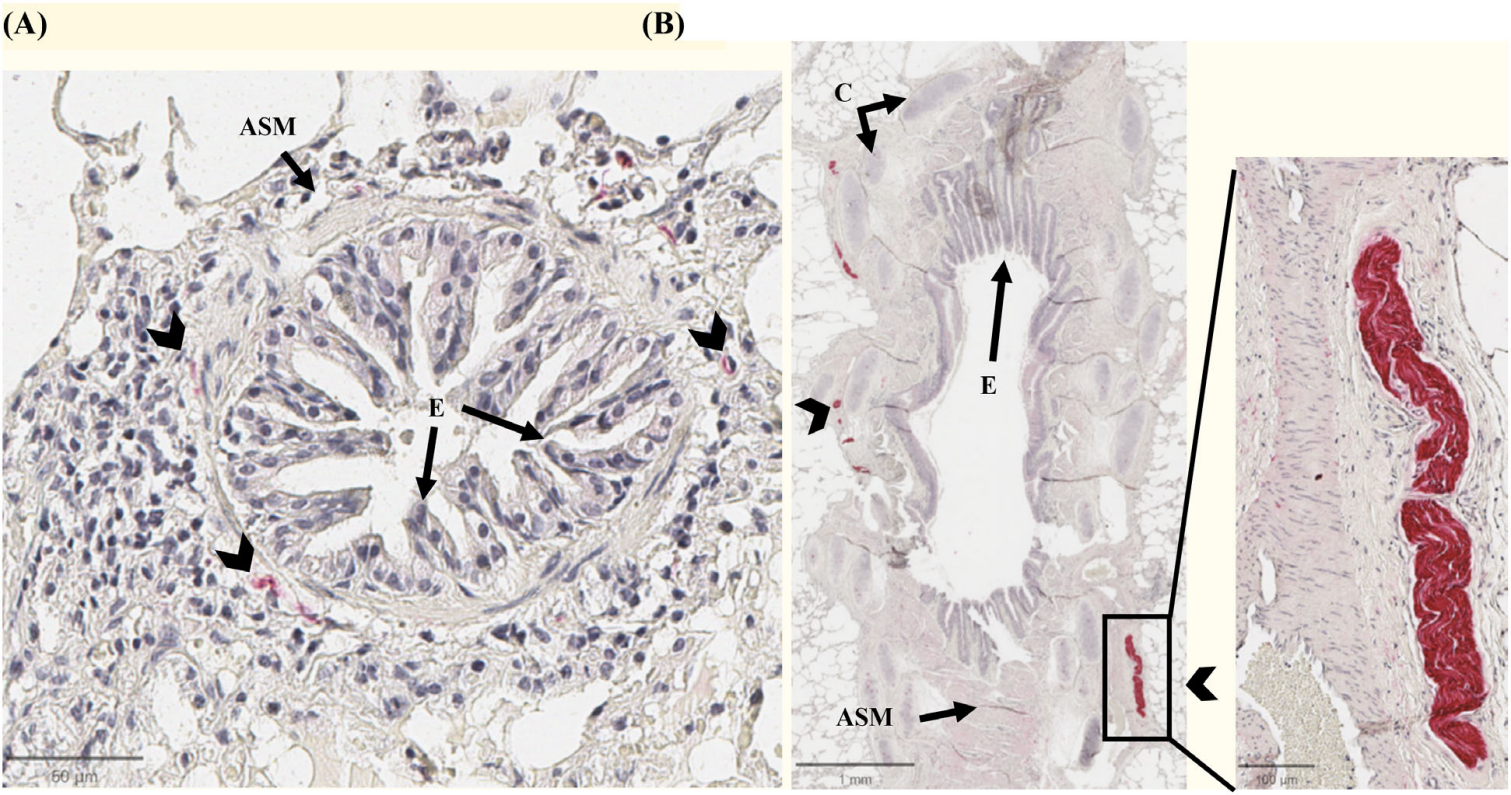
Immunohistochemistry of peribronchial innervation. Representative images of the peribronchial innervation of a horse with asthma stained with anti‐s100 antibody (Vector Red, counterstained with hematoxylin). In panel A, small nerves (arrowheads) around a bronchiole. Panel B shows a large nerve in the surrounding of a bronchi. ASM, airway smooth muscle; C, cartilage; E, bronchi epithelium.

The protocol was adapted from a previous study using equine tissue.^23^ The lung tissue was fixed in 4% formaldehyde for 24 hours and embedded in paraffin. Five‐micrometer histologic slides were treated with citrate‐based antigen unmasking solution (Vector Laboratories, #H‐3300, Burlingame, California) and incubated in blocking solution (phosphate buffered saline with 10% goat serum [Vector Laboratories, #S‐1000, Burlingame, California]) for 1 hour. The slides were incubated overnight at 4°C with a rabbit polyclonal anti‐s100 antibody (dilution 1:2500, Dako #IR504, Agilent Technologies, Mississauga, Ontario, Canada), a neural marker for Schwann cells. Negative controls were processed with the same protocol using an isotype control antibody (dilution 1:2500, Rabbit IgG, Thermofisher Scientific, #02‐6102, Burlington, Ontario, Canada) instead of the primary antibody. Equine intestinal tissue was used as a positive control. Slides were incubated for 45 minutes at room temperature with a goat anti‐rabbit biotinylated secondary antibody (dilution 1:200, Jackson Immunoresearch Laboratory, #B8895, West Grove, Pennsylvania) and developed with Vecastain ABC‐alkaline phosphatase kit (Vector Laboratories, #SK5100, Burlingame, California) to mark the neural tissue in red (Figure 1A,B). Samples were then counterstained with Harry's hematoxylin and mounted in Leica Micromount (Surgipath Micromount Mounting Medium, Leica #3801731, Buffalo Grove, Illinois).

### 2.4 Histomorphometric analysis

**FIGURE 2:**
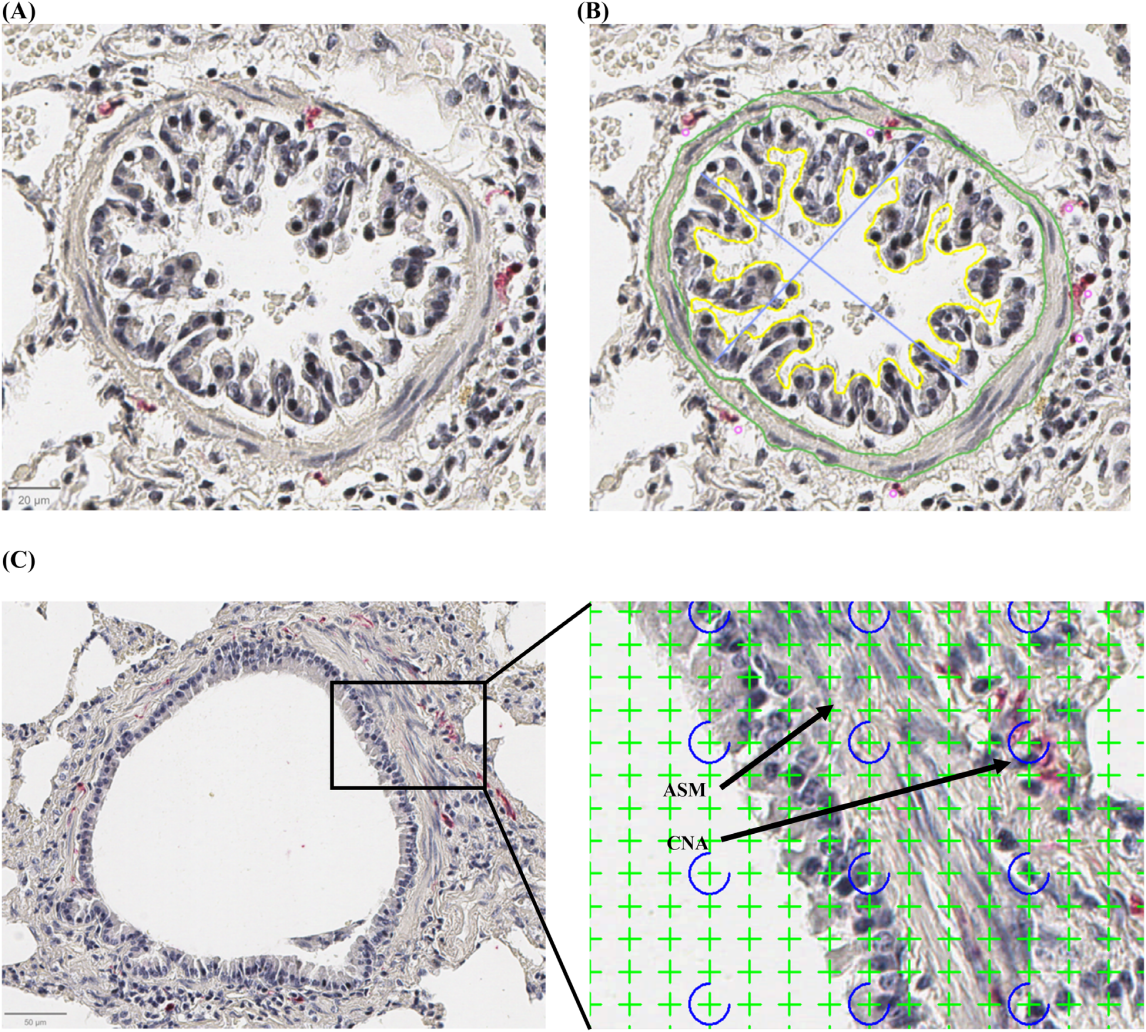
Histomorphometric measurements by tracing and point counting. (A,B) Bronchiole (histological section, ×40). IP = internal perimeter (yellow), LA = lumen area (area inside the yellow outline), ID = internal diameters (blue lines), ASM = smooth muscle area (areas between the green lines), NBN = number of peribronchial nerves (pink dots). (C) Point counting technique using a grid of 1024 crosses per screen. Crosses were counted as either nerve (CNA, cumulative nerve area) if they encompassed red‐stained peribronchial tissue or as smooth muscle (ASM, smooth muscle area).

Histologic slides were scanned and digitalized at ×40 magnification as SVS images. Histomorphometry was performed using morphometric tracing with QuPath software (version 0.4.2) and point counting analysis with newCAST software (Visiopharm, version 2019.02.16005, Hoersholm, Hovedstaden, DK), as previously described.^24^, ^25^ Four to 8 peripheral bronchioles and 2 to 5 peripheral pulmonary arteries with a minimal internal diameter of 40 μm were analyzed for each horse. The internal diameter (ID), internal perimeter (IP), lumen area (LA), number of peribronchial nerves (NBN), and number of smooth‐muscle associated nerves (NBN‐ASM) were measured using morphometric tracing (Figure 2A). Because the nerve area was challenging to measure with morphometric tracing because of the tortuous appearance and small size, point counting analysis was used to measure the cumulative nerve surface area (CNA) and smooth muscle‐associated cumulative nerve surface area (CNA‐ASM; Figure 2B). Nerves were considered to be within the peribronchial or perivascular region if the proximity was closest to bronchi or arteries, respectively, as opposed to alternative structures. Smooth muscle‐associated nerves were defined as those within or lining the airway smooth muscle. Airway and vascular smooth muscle area (ASM and VSM) were measured both by morphometric tracing (MT) and point counting analysis (PC). Each point corresponding to an area of 81.29 μm^2^, nerve and smooth muscle area were estimated as n points × 81.29 μm^2^. Peripheral bronchi were included if they had an internal diameter of less than 2 mm, a major‐to‐minor axis ratio below 2 (Figure 2), and an intact epithelium. Measurements were corrected by the internal perimeter squared to allow comparison between airways of different sizes.^26^ The internal diameter, perimeter, lumen area, number of nerves (NVN), and vascular smooth muscle area (VSM) were measured in pulmonary arteries by morphometric tracing (QuPath). Arteries were included if the internal diameter was greater than 40 μm, the major‐to‐minor axis ratio was less than 3, and the endothelium was intact. The definition of measured variables and measurement methods are summarized in Table 1.

**TABLE 1:** Histomorphometric measurements.

Linear measurements (1D) (μm)		
Internal perimeter (IP)	MT	Outline of the internal epithelium
Internal diameter (ID)	MT	Longest distance between epitheliums
Number of peribronchial nerves (NBN)	MT	Number of nerves in the peribronchial region
Number of perivascular nerves (NVN)	MT	Number of nerves in the perivascular region
Number of smooth muscle associated nerves (NBN‐ASM)	MT	Number of nerves within or lining the airway smooth muscle

Bidimensional measurements (2D) (μm^2^)		
Lumen area (LA)	MT	Area enclosed by the internal perimeter
Airway smooth muscle area (ASM‐MT)	MT	Sum of smooth muscle area measured by tracing the border of airway smooth muscle bundles
Vascular smooth muscle area (VSM)	MT	Sum of smooth muscle area measured by tracing the border of vascular smooth muscle bundles
Airway smooth muscle area (ASM‐PC)	PC	Sum of smooth muscle area measured by tracing the border of airway smooth muscle bundles
Cumulative nerve area (CNA)	PC	Sum of nerve‐positive points × 81.29 μm^2^
Airway smooth muscle associated nerve area (CNA‐ASM)	PC	Sum of smooth muscle‐associated nerve‐positive points × 81.29 μm^2^

Abbreviations: MT, morphometric tracing; PC, point counting analysis.

### 2.5 Statistical analysis

The sample size was estimated based on other airway and vascular remodeling studies in horses with severe asthma.^20^, ^27^ Data normality was assessed by visual analysis and with Shapiro‐Wilk tests. Descriptive data were expressed as the median and interquartile range (IQR). Comparisons between controls and horses with asthma were performed using the Mann‐Whitney test. Horses in remission and exacerbation were grouped for histomorphometric data (n = 8 controls and 8 horses with severe asthma) to increase statistical power and because no difference were expected (nor observed). Only horses in exacerbation were analyzed when comparing lung function and BALF neutrophil percentages (n = 8 controls and 5 horses with severe asthma in exacerbation) as there were too few horses in remission (n = 3) to perform appropriate statistical analysis. Correlations between histomorphometric data and pulmonary function and BALF neutrophils were evaluated with Spearman's correlations. Differences were considered significant at *P* < .05. Statistical analysis was performed using GraphPad Prism version 9.2.0 (GraphPad Software, San Diego, California).

## 3 RESULTS

### 3.1 Horses

Sixteen horses were studied (14 mares, 2 geldings). Age was significantly higher in the severe asthma group (median [IQR]: 16.0 years old [3.8]) than in the control group (9.0 years old [7.0], *P* = .04). Weight was not statistically different between both groups (asthma group: 511.5 ± 116.5 kg; controls: 475.0 ± 71.5 kg [*P* = .49]).

### 3.2 Lung function and airway inflammation

As expected, horses with severe exacerbation of asthma had significantly higher *R*_*L*_, *E*_*L*,_ and BALF neutrophilia when compared with controls (Figures S1A,B). A comparison of controls and horses in exacerbation with horses in remission was not performed because of the small sample size of the latter group.

### 3.3 Histomorphometric analysis

#### 3.3.1 Airways (NBN)

**FIGURE 3:**
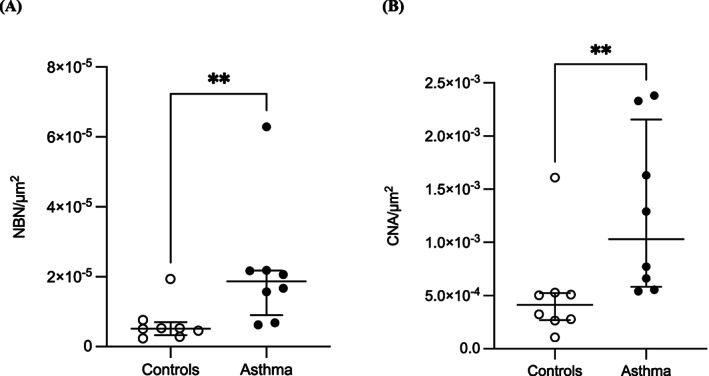
Airway innervation. (A) Number of nerves (NBN) and (B) cumulative nerve area (CNA) corrected for the internal perimeter squared (μm^2^) in the peribronchial region of horses with asthma (n = 8) and controls (n = 8). Horizontal lines represent the median, and the error bars represent the interquartile range.

The number of nerves corrected for the perimeter squared was significantly higher in horses with asthma (median [IQR]: 1.87 × 10^−5^ nerves/μm^2^ [1.28 × 10^−5^]) compared with controls (5.17 × 10^−6^ nerves/μm^2^ [3.76 × 10^−6^], *P* = .01, Figure 3A). Additionally, the cumulative nerve area of peribronchial innervation was also larger in horses with severe asthma (1.03 × 10^−3^ CNA/μm^2^ [1.57 × 10^−3^]) when compared with controls (4.14 × 10^−4^ CNA/μm^2^ [2.54 × 10^−4^], *P* = .01, Figure 3B).

**FIGURE 4:**
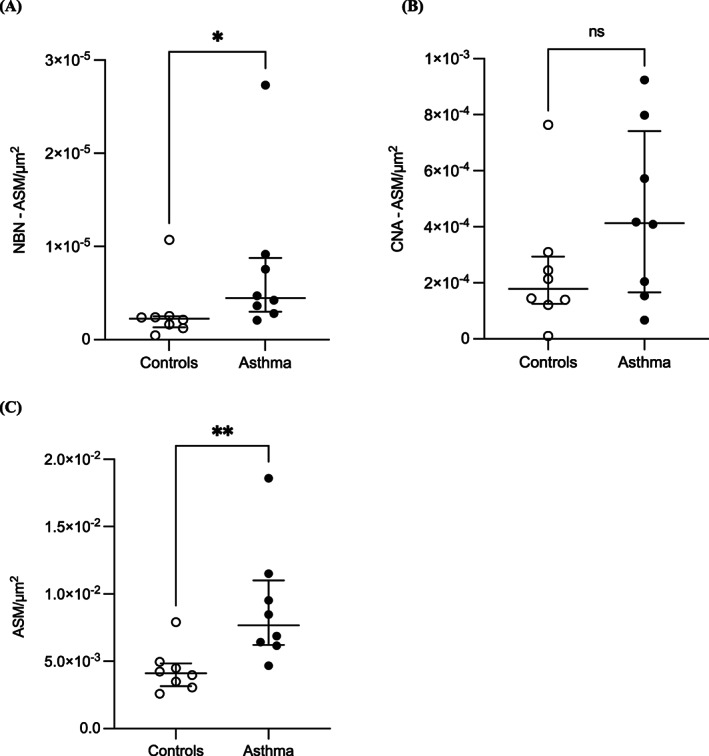
Smooth muscle‐associated innervation. (A) Number of nerves (NBN‐ASM) and (B) cumulative nerve area (CNA‐ASM) within or lining airway smooth muscle (ASM) corrected for the internal perimeter squared (μm^2^) in the peribronchial region of horses with asthma (n = 8) and controls (n = 8). (C) Airway smooth muscle area (ASM; μm^2^) of horses with asthma (n = 8) and controls (n = 8). Horizontal lines represent the median, and the error bars represent the interquartile range.

The number of smooth muscle‐associated nerves was also higher in horses with asthma (asthma group: 4.47 × 10^−6^ nerves/μm^2^ (5.75 × 10^−6^); control group: 2.26 × 10^−6^ nerves/μm^2^ (1.16 × 10^−6^), *P* = .03, Figure 4A). The smooth muscle‐associated cumulative nerve area was not significantly higher in horses with asthma (asthma group: 4.13 × 10^−4^ CNA/μm^2^ [5.75 × 10^−4^]; control group: 1.79 × 10^−4^ CNA/μm^2^ [1.68 × 10^−4^], *P* = .16, Figure 4B). Airway smooth muscle area (ASM) was larger in horses with severe asthma compared with healthy horses (asthma group: 7.68 × 10^−3^ ASM/μm^2^ [4.80 × 10^−3^]; control group, 4.11 × 10^−3^ ASM/μm^2^ [1.68 × 10^−3^], *P* = .01, Figure 4C). The correlation between airway smooth muscle area measured by both histomorphometric techniques (morphometric tracing and point counting analysis) was strong (Spearman's correlation; *r* = .97, *P* < .001) and is presented in Figure S3.

Airway innervation (number of nerves and cumulative nerve area) in horses in exacerbation was not correlated with lung function (resistance and elastance: Spearman's correlation—*r* < .35, *P* > .5). Age was not correlated with the number of nerves or the cumulative nerve area within each group (Spearman's correlation—asthma group: *r* = .09 [*P* = .83] and *r* = .39 [*P* = .34] for the number of nerves and the cumulative nerve area, respectively); control group: *r* = .56 (*P* = .15) and *r* = .47 (*P* = .24) for the number of nerves and the cumulative nerve area, respectively.

#### 3.3.2 Pulmonary arteries (NVN)

The number of nerves was not significantly different in horses with asthma (2.87 × 10^−5^ nerves/μm^2^ [2.35 × 10^−5^]) compared with controls (1.31 × 10^−5^ nerves/μm^2^ [6.67 × 10^−5^], *P* = .57, Figure S4A). Vascular smooth muscle area (VSM) was increased in horses with severe asthma compared with controls (asthma group: 3.97 × 10^−2^ VSM/μm^2^ (5.43 × 10^−2^); control group, 2.47 × 10^−2^ VSM/μm^2^ (2.68 × 10^−2^), *P* = .04, 1‐tailed test, Figure S4B).

## 4 DISCUSSION

### 4.1 Peripheral airway innervation and asthma

In the present study, the peribronchial innervation of horses with asthma was characterized with immunohistochemistry and histomorphometry. The results indicate an increase in peribronchial nerve supply of the peripheral airways in horses with severe asthma. Those results could be interpreted as (i) an increased number of nerves without an increase in size or (ii) an increase in both the number and the size of the nerves. The results do not allow us to conclude on those theories, as the point‐counting method can only estimate the *cumulative* area of the nerves, and not the size of individual nerves.

Airway and lung parenchymal nerves supply the airway smooth muscle, glands, epithelium, and vascular system.^12^, ^13^ Alteration of the structure or the function of airway innervation can therefore contribute to impaired smooth muscle tone, modulation of inflammation, and mucus secretion.^10^ By mediating local airway reflexes and releasing neurotransmitters such as substance P and calcitonin gene‐related peptide, afferent sensory nerves play a major role in inducing airway smooth muscle contraction in other species.^9^ Increased cholinergic tone also contributes, in part, to bronchoconstriction and mucus hypersecretion, hence the effectiveness of anticholinergics as bronchodilators in horses with asthma.^28^, ^29^, ^30^ Furthermore, the persistence of AHR in the absence of inflammation during the remission state of asthma suggests that other mechanisms, such as neural activity, are involved.^7^, ^31^, ^32^ Several studies reported on the role of airway nerves in the development of AHR, as evidenced by the inhibition of AHR in response to stimuli after anticholinergic administration.^7^, ^31^ Moreover, AHR is inhibited by vagotomy in dogs and mice,^14^, ^33^ demonstrating the importance of neural activity in the pathophysiology of asthma. Functional alterations of airway innervation in asthma have also been shown in horses. Derksen et al investigated the role of vagal input on ovalbumin‐sensitized ponies. They found that vagal blockade reversed the increased minute ventilation, lung resistance, and respiratory rate and increased the tidal volume.^21^ These results suggest that vagal fiber activity and sensitivity could be involved in the pathogenesis of airway obstruction in horses with asthma.

### 4.2 Airway smooth muscle remodeling and innervation

In agreement with previous reports, airway smooth muscle area was significantly increased in horses with severe asthma in the current study.^18^, ^19^, ^34^ The increase in nerves associated with smooth muscle in horses with asthma supports the theory that innervation is linked to smooth muscle remodeling. However, increased peribronchial smooth muscle‐associated innervation could be either a cause or a consequence of the smooth muscle remodeling observed in equine asthma or a combination of both. In utero, the maturation of airway innervation follows the development of smooth muscle^35^ and an increased innervation could be needed to compensate for the increased number of muscle cells (hyperplasia) occurring in asthma.^19^ Therefore, smooth muscle remodeling could precede the augmentation of innervation. On the other hand, there is evidence that sympathetic innervation can induce and promote vascular smooth muscle differentiation,^36^ which could also affect smooth muscle in the airway. However, sympathetic innervation plays a limited role in regulating airway caliber, and, at least in humans, sympathetic nerves do not innervate ASM directly.^10^ Finally, studies have shown that anticholinergics reduce bronchial muscle mass, which could indicate smooth muscle remodeling is at least partly caused by altered innervation. For example, the administration of tiotropium bromide, an anticholinergic drug, reduced the airway smooth muscle mass and thickness, contractility, and myosin expression in murine models of asthma.^37^, ^38^ Of note, 1 of the control horses was an outlier in the airway innervation and airway smooth muscle data, but met all the clinical, lung function and lung inflammation criteria. This might suggest (i) increased airway smooth muscle leads to increased airway innervation, regardless of the asthma status, or (ii) this specific horse could have had mild or moderate asthma that went undetected with standard lung function, BAL cytology and physical examination at rest.

### 4.3 Airway inflammation and innervation

In addition to the effects on ASM hyperplasia/hypertrophy, peribronchial nerves can contribute to pulmonary inflammation as inflammatory cells express muscarinic receptors and are susceptible to the effect of acetylcholine released by efferent motor airway nerves.^13^, ^32^ Conversely, the effects of inflammatory cells on airway nerve remodeling have also been reported.^39^, ^40^ In fact, the release of acetylcholine at the neuromuscular junction, as well as the sensitivity of smooth muscle to acetylcholine, could be increased by inflammatory mediators present in horses' airways.^22^ Moreover, inflammatory mediators such as histamine and leukotriene increased endogenous cholinergic tone in peripheral and central airways of horses.^22^, ^41^ Therefore, airway innervation could contribute to airway inflammation but also be affected by it, not only functionally but also structurally.

### 4.4 Vascular smooth muscle remodeling and innervation

The increase in vascular smooth muscle area in horses with severe asthma is consistent with a previous study.^20^ In a murine model of asthma, both ASM and VSM remodeling occurred and persisted at least 1 month after allergen exposure.^42^ The lack of increased innervation in the pulmonary arteries of horses suggests (a) the increased peribronchial innervation is specific to the airways and (b) the increase in VSM is not secondary to increased innervation. This does not allow to conclude on the mechanisms of the increased airway innervation, but it makes a response to circulating cytokines less likely. Also, vascular smooth muscle remodeling can be caused by other mechanisms, such as chronic hypoxia and inflammation, that can induce migration and proliferation.^43^, ^44^, ^45^ The low statistical power could also contribute to the lack of observed increased pulmonary artery innervation as a post hoc power calculation shows a statistical power of less than 80% with the data obtained.

### 4.5 Immunohistochemistry and histomorphometry

Antibodies targeting s100 proteins are a general neural marker for the s100 calcium‐binding protein, which is present in the cytoplasm and nucleus of Schwann cells peripheral neurons.^23^, ^46^ It is also used as a neural marker in the human superficial fascia, temporal dura mater, epidermis, and other tissues.^46^, ^47^, ^48^ However, it does not allow discrimination between autonomic and sensory nerve fibers. The use of specific markers for neuropeptides released from afferent sensory nerves, such as substance P, calcitonin gene‐related peptide, or neurokinin A could provide more information regarding functional neural activity in future studies. Protein Gene Product 9.5 is commonly used in human literature as a pan‐neural marker.^49^, ^50^ However, immunohistochemical staining with this antibody was deemed poor and inadequate for histological quantification in the present study (unpublished data). Two histomorphometric methods were used to characterize peribronchial innervation and smooth muscle remodeling in this study. While morphometric tracing allowed for straightforward manual counting of peribronchial nerves, the cumulative nerve area could only be assessed via point‐counting analysis because of the tortuous and small nature of the nerve fibers. Previous studies investigating airway remodeling in severe equine asthma also combined these 2 histomorphometric approaches.^20^, ^51^

### 4.6 Limitations

Several limitations of this study should be acknowledged. First, only a small sample size was included based on tissue availability. For the same reason, we studied tissues from horses in either remission or exacerbation of asthma. However, horses in asthma remission had been in exacerbation less than a year before tissue sampling. Because structural plasticity of airway innervation appears to be a chronic phenomenon,^10^ and the horses with severe asthma we study had been affected for at least a year, it seems appropriate to have grouped these samples. Additionally, all samples were collected from the caudodorsal lung field and may not represent changes occurring in other regions of the lung. In a study assessing pulmonary arteries remodeling in equine asthma, changes were mainly located in the apical and caudodorsal lung.^20^ Of note, horses with milder forms of asthma were not included in this study; therefore, extrapolation to this population is not possible. The quantification of the nerve architecture can be challenging as quantitative manual analyses are susceptible to sampling errors. Studies in humans have generated inconsistent results with this technique, and it was suggested that bronchi originating from different airway locations could have distinct nerve structures.^52^, ^53^, ^54^ While not evaluated in the present study, recent findings indicate that the central and peripheral airways are innervated to a comparable degree.^55^, ^56^ Finally, histomorphometric quantification of rare events such as nerve fibers faces multiple challenges, including overestimating size (the result of the events counted and the size of the points [ie, crosses in Figure 2B]). Thus, the findings regarding cumulative nerve area are helpful for comparing groups but should not be interpreted as absolute nerve size.

## 5 CONCLUSION

This study showed an increased innervation of the peripheral airways in horses with severe asthma, which could contribute to asthma severity. To the knowledge of the authors, this is the first study investigating histologic airway innervation in equine asthma. Bronchial innervation dysfunction could be involved in the initiation and persistence of severe equine asthma.

## CONFLICT OF INTEREST DECLARATION

Authors declare no conflict of interest.

## OFF‐LABEL ANTIMICROBIAL DECLARATION

Authors declare no off‐label use of antimicrobials.

## INSTITUTIONAL ANIMAL CARE AND USE COMMITTEE (IACUC) OR OTHER APPROVAL DECLARATION

Horses entered the Equine Respiratory Tissue Biobank under the animal use and care protocols associated with the projects they were enrolled in before euthanasia (Rech‐1578).

## HUMAN ETHICS APPROVAL DECLARATION

Authors declare human ethics approval was not needed for this study.

## Supporting information


**Data S1.** Supporting Information Figures.

## References

[1] Hotchkiss JW , Reid SWJ , Christley RM . A survey of horse owners in Great Britain regarding horses in their care. Part 2: risk factors for recurrent airway obstruction. Equine Vet J. 2007;39(4):301‐308. doi:10.2746/042516407X180129 17722720

[2] Couetil L , Cardwell JM , Leguillette R , et al. Equine asthma: current understanding and future directions. Front Vet Sci. 2020;7:450. doi:10.3389/fvets.2020.00450 32903600 PMC7438831

[3] Robinson NE , Chairperson W . International workshop on equine chronic airway disease Michigan State University 16–18 June 2000. Equine Vet J. 2001;33(1):5‐19. doi:10.2746/042516401776767412 11191611

[4] Leclere M , Lavoie‐Lamoureux A , Lavoie JP . Heaves, an asthma‐like disease of horses. Respirology. 2011;16(7):1027‐1046. doi:10.1111/j.1440-1843.2011.02033.x 21824219

[5] Leclere M , Lavoie‐Lamoureux A , Joubert P , et al. Corticosteroids and antigen avoidance decrease airway smooth muscle mass in an equine asthma model. Am J Respir Cell Mol Biol. 2012;47(5):589‐596. doi:10.1165/rcmb.2011-0363OC 22721832

[6] Polverino M , Polverino F , Fasolino M , Andò F , Alfieri A , De Blasio F . Anatomy and neuro‐pathophysiology of the cough reflex arc. Multidiscip Respir Med. 2012;7(1):5. doi:10.1186/2049-6958-7-5 22958367 PMC3415124

[7] Canning BJ , Woo A , Mazzone SB . Neuronal modulation of airway and vascular tone and their influence on nonspecific airways responsiveness in asthma. J Allergy. 2012;2012:1‐7. doi:10.1155/2012/108149 PMC3485909 23150736

[8] Lommatzsch M . Airway hyperresponsiveness: new insights into the pathogenesis. Semin Respir Crit Care Med. 2012;33(6):579‐587. doi:10.1055/s-0032-1325617 23047309

[9] Verhein KC , Fryer AD , Jacoby DB . Neural control of airway inflammation. Curr Allergy Asthma Rep. 2009;9(6):484‐490. doi:10.1007/s11882-009-0071-9 19814922

[10] Kistemaker LEM , Prakash YS . Airway innervation and plasticity in asthma. Physiology (Bethesda). 2019;34(4):283‐298. doi:10.1152/physiol.00050.2018 31165683 PMC6863372

[11] Belvisi MG . Airway sensory innervation as a target for novel therapies: an outdated concept? Curr Opin Pharmacol. 2003;3(3):239‐243. doi:10.1016/S1471-4892(03)00048-1 12810186

[12] Kistemaker LEM , Bos ST , Mudde WM , et al. Muscarinic M3 receptors contribute to allergen‐induced airway remodeling in mice. Am J Respir Cell Mol Biol. 2014;50(4):690‐698. doi:10.1165/rcmb.2013-0220OC 24156289

[13] Kistemaker LEM , Gosens R . Acetylcholine beyond bronchoconstriction: roles in inflammation and remodeling. Trends Pharmacol Sci. 2015;36(3):164‐171. doi:10.1016/j.tips.2014.11.005 25511176

[14] Liu R , Song J , Li H , et al. Treatment of canine asthma by high selective vagotomy. J Thorac Cardiovasc Surg. 2014;148(2):683‐689. doi:10.1016/j.jtcvs.2013.12.041 24521967

[15] Ollerenshaw SL , Jarvis D , Sullivan CE , Woolcock AJ . Substance P immunoreactive nerves in airways from asthmatics and nonasthmatics. Eur Respir J. 1991;4(6):673‐682. 1716217

[16] Mazzone SB , Undem BJ . Vagal afferent innervation of the airways in health and disease. Physiol Rev. 2016;96(3):975‐1024. doi:10.1152/physrev.00039.2015 27279650 PMC4982036

[17] Matera MG , Amorena M , Lucisano A . Innervation of equine airways. Pulm Pharmacol Ther. 2002;15(6):503‐511. doi:10.1006/pupt.2002.0390 12493337

[18] Herszberg B , Ramos‐Barbón D , Tamaoka M , Martin JG , Lavoie JP . Heaves, an asthma‐like equine disease, involves airway smooth muscle remodeling. J Allergy Clin Immunol. 2006;118(2):382‐388. doi:10.1016/j.jaci.2006.03.044 16890762

[19] Leclere M , Lavoie‐Lamoureux A , Gélinas‐Lymburner É , David F , Martin JG , Lavoie JP . Effect of antigenic exposure on airway smooth muscle remodeling in an equine model of chronic asthma. Am J Respir Cell Mol Biol. 2011;45(1):181‐187. doi:10.1165/rcmb.2010-0300OC 20935189

[20] Ceriotti S , Bullone M , Leclere M , Ferrucci F , Lavoie JP . Severe asthma is associated with a remodeling of the pulmonary arteries in horses. PloS One. 2020;15(10):e0239561. doi:10.1371/journal.pone.0239561 33091038 PMC7580920

[21] Derksen FJ , Robinson NE , Slocombe RF . Ovalbumin‐induced lung disease in the pony: role of vagal mechanisms. J Appl Physiol Respir Environ Exerc Physiol. 1982;53(3):719‐725. doi:10.1152/jappl.1982.53.3.719 7129995

[22] Olszewski MA , Zhang XY , Robinson NE . Pre‐ and postjunctional effects of inflammatory mediators in horse airways. Am J Physiol – Lung Cell Mol Physiol. 1999;277(2):L327‐L333. doi:10.1152/ajplung.1999.277.2.L327 10444527

[23] Pujol R , Girard CA , Richard H , et al. Synovial nerve fiber density decreases with naturally‐occurring osteoarthritis in horses. Osteoarthr Cartil. 2018;26(10):1379‐1388. doi:10.1016/j.joca.2018.06.006 29958917

[24] Gundersen HJG , Bendtsen TF , Korbo L , et al. Some new, simple and efficient stereological methods and their use in pathological research and diagnosis. APMIS. 1988;96(1‐6):379‐394. doi:10.1111/j.1699-0463.1988.tb05320.x 3288247

[25] Bankhead P , Loughrey MB , Fernández JA , et al. QuPath: open source software for digital pathology image analysis. Sci Rep. 2017;7(1):16878. doi:10.1038/s41598-017-17204-5 29203879 PMC5715110

[26] James AL , Hogg JC , Dunn LA , Paré PD . The use of the internal perimeter to compare airway size and to calculate smooth muscle shortening. Am Rev Respir Dis. 1988;138(1):136‐139. doi:10.1164/ajrccm/138.1.136 3202392

[27] Millares‐Ramirez EM , Lavoie JP . Bronchial angiogenesis in horses with severe asthma and its response to corticosteroids. J Vet Intern Med. 2021;35(4):2026‐2034. doi:10.1111/jvim.16159 34048095 PMC8295704

[28] Canning BJ . Reflex regulation of airway smooth muscle tone. J Appl Physiol. 2006;101(3):971‐985. doi:10.1152/japplphysiol.00313.2006 16728519

[29] de Lagarde M , Rodrigues N , Chevigny M , Beauchamp G , Albrecht B , Lavoie JP . N‐butylscopolammonium bromide causes fewer side effects than atropine when assessing bronchoconstriction reversibility in horses with heaves. Equine Vet J. 2014;46(4):474‐478. doi:10.1111/evj.12229 24423012

[30] Couetil L , Hammer J , Miskovic Feutz M , Nogradi N , Perez‐Moreno C , Ivester K . Effects of N‐butylscopolammonium bromide on lung function in horses with recurrent airway obstruction. J Vet Intern Med. 2012;26(6):1433‐1438. doi:10.1111/j.1939-1676.2012.00992.x 22925156

[31] Santing RE , Pasman Y , Olymulder C , Roffel AF , Meurs H , Zaagsma J . Contribution of a cholinergic reflex mechanism to allergen‐induced bronchial hyperreactivity in permanently instrumented, unrestrained Guinea‐pigs. Br J Pharmacol. 1995;114(2):414‐418. 7881742 10.1111/j.1476-5381.1995.tb13242.x PMC1510260

[32] Lutz W , Sułkowski WJ . Vagus nerve participates in regulation of the airways: inflammatory response and hyperreactivity induced by occupational asthmogens. Int J Occup Med Environ Health. 2004;17(4):417‐431. 15852756

[33] McAlexander MA , Gavett SH , Kollarik M , Undem BJ . Vagotomy reverses established allergen‐induced airway hyperreactivity to methacholine in the mouse. Respir Physiol Neurobiol. 2015;212‐214:20‐24. doi:10.1016/j.resp.2015.03.007 PMC4827162 25842220

[34] Bullone M , Beauchamp G , Godbout M , Martin JG , Lavoie JP . Endobronchial ultrasound reliably quantifies airway smooth muscle remodeling in an equine asthma model. PLoS One. 2015;10(9):e0136284. doi:10.1371/journal.pone.0136284 26348727 PMC4562526

[35] Sparrow MP , Lamb JP . Ontogeny of airway smooth muscle: structure, innervation, myogenesis and function in the fetal lung. Respir Physiol Neurobiol. 2003;137(2‐3):361‐372. doi:10.1016/s1569-9048(03)00159-9 14516738

[36] Damon DH . Sympathetic innervation promotes vascular smooth muscle differentiation. Am J Physiol Heart Circ Physiol. 2005;288(6):H2785‐H2791. doi:10.1152/ajpheart.00354.2004 15665063

[37] Gosens R , Bos IST , Zaagsma J , Meurs H . Protective effects of tiotropium bromide in the progression of airway smooth muscle remodeling. Am J Respir Crit Care Med. 2005;171(10):1096‐1102. doi:10.1164/rccm.200409-1249OC 15695490

[38] Ohta S , Oda N , Yokoe T , et al. Effect of tiotropium bromide on airway inflammation and remodelling in a mouse model of asthma. Clin Exp Allergy. 2010;40(8):1266‐1275. doi:10.1111/j.1365-2222.2010.03478.x 20337647

[39] Drake MG , Scott GD , Blum ED , et al. Eosinophils increase airway sensory nerve density in mice and in human asthma. Sci Transl Med. 2018;10(457):eaar8477. doi:10.1126/scitranslmed.aar8477 30185653 PMC6592848

[40] Drake MG , Lebold KM , Roth‐Carter QR , et al. Eosinophil and airway nerve interactions in asthma. J Leukoc Biol. 2018;104(1):61‐67. doi:10.1002/JLB.3MR1117-426R 29633324 PMC6541210

[41] Olszewski MA , Robinson NE , Derksen FJ . In vitro responses of equine small airways and lung parenchyma. Respir Physiol. 1997;109(2):167‐176. doi:10.1016/S0034-5687(97)00053-4 9299648

[42] Rydell‐Törmänen K , Uller L , Erjefält JS . Allergic airway inflammation initiates long‐term vascular remodeling of the pulmonary circulation. Int Arch Allergy Immunol. 2009;149(3):251‐258. doi:10.1159/000199721 19218818

[43] Lee J , Kang H . Hypoxia promotes vascular smooth muscle cell proliferation through microRNA‐mediated suppression of cyclin‐dependent kinase inhibitors. Cell. 2019;8(8):802. doi:10.3390/cells8080802 PMC6721514 31370272

[44] Crosswhite P , Sun Z . Molecular mechanisms of pulmonary arterial remodeling. Mol Med. 2014;20(1):191‐201. doi:10.2119/molmed.2013.00165 24676136 PMC4002851

[45] Harkness LM , Kanabar V , Sharma HS , Westergren‐Thorsson G , Larsson‐Callerfelt AK . Pulmonary vascular changes in asthma and COPD. Pulm Pharmacol Ther. 2014;29(2):144‐155. doi:10.1016/j.pupt.2014.09.003 25316209

[46] Sloekers JCT , Herrler A , Hoogland G , et al. Nerve fiber density differences in the temporal dura mater: an explanation for headache after temporal lobectomy? An anatomical study. J Chem Neuroanat. 2022;121:102082. doi:10.1016/j.jchemneu.2022.102082 35158040

[47] Kawakami T , Ishihara M , Mihara M . Distribution density of intraepidermal nerve fibers in normal human skin. J Dermatol. 2001;28(2):63‐70. doi:10.1111/j.1346-8138.2001.tb00091.x 11320708

[48] Fede C , Petrelli L , Pirri C , et al. Innervation of human superficial fascia. Front Neuroanat. 2022;16:981426. doi:10.3389/fnana.2022.981426 36106154 PMC9464976

[49] Bradbury JM , Thompson RJ . Immunoassay of the neuronal and neuroendocrine marker PGP 9.5 in human tissues. J Neurochem. 1985;44(2):651‐653. doi:10.1111/j.1471-4159.1985.tb05461.x 2856932

[50] Thompson RJ , Doran JF , Jackson P , Dhillon AP , Rode J . PGP 9.5—a new marker for vertebrate neurons and neuroendocrine cells. Brain Res. 1983;278(1‐2):224‐228. doi:10.1016/0006-8993(83)90241-x 6640310

[51] Bullone M , Vargas A , Elce Y , Martin JG , Lavoie JP . Fluticasone/salmeterol reduces remodelling and neutrophilic inflammation in severe equine asthma. Sci Rep. 2017;7(1):8843. doi:10.1038/s41598-017-09414-8 28821845 PMC5562887

[52] Howarth PH , Springall DR , Redington AE , Djukanovic R , Holgate ST , Polak JM . Neuropeptide‐containing nerves in endobronchial biopsies from asthmatic and nonasthmatic subjects. Am J Respir Cell Mol Biol. 1995;13(3):288‐296. doi:10.1165/ajrcmb.13.3.7654385 7654385

[53] Howarth PH , Djukanovic R , Wilson JW , Holgate ST , Springall DR , Polak JM . Mucosal nerves in endobronchial biopsies in asthma and non‐asthma. Int Arch Allergy Appl Immunol. 1991;94(1‐4):330‐333. doi:10.1159/000235396 1718895

[54] Chanez P , Springall D , Vignola AM , et al. Bronchial mucosal immunoreactivity of sensory neuropeptides in severe airway diseases. Am J Respir Crit Care Med. 1998;158(3):985‐990. doi:10.1164/ajrccm.158.3.9608104 9731035

[55] Scott GD , Blum ED , Fryer AD , Jacoby DB . Tissue optical clearing, three‐dimensional imaging, and computer morphometry in whole mouse lungs and human airways. Am J Respir Cell Mol Biol. 2014;51(1):43‐55. doi:10.1165/rcmb.2013-0284OC 24471696 PMC4091855

[56] West PW , Canning BJ , Merlo‐Pich E , Woodcock AA , Smith JA . Morphologic characterization of nerves in whole‐mount airway biopsies. Am J Respir Crit Care Med. 2015;192(1):30‐39. doi:10.1164/rccm.201412-2293OC 25906337 PMC4511424

