# Supplementary material for: Severe asthma in horses is associated with increased airway innervation
Source: J Vet Intern Med. 2023 Dec 6;38(1):485–94. doi: 10.1111/jvim.16941 (PMC10800206; doi:10.1111/jvim.16941)

Supplemental Material

Fig S1. Lung function. A) Pulmonary resistance (R_L) and B) pulmonary elastance (E_L) in horses with asthma (black dots) in exacerbation (n = 5), in remission (n = 3), and controls (white dots, n = 8) before euthanasia. Horizontal lines represent the median, and the error bars represent the interquartile range. Horses in remission were excluded from the statistical analysis.

A

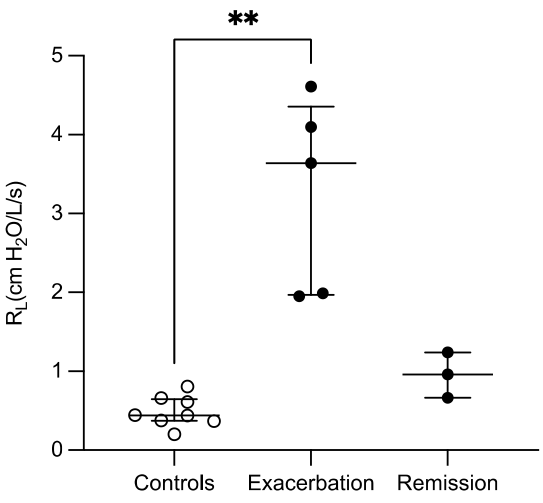

B

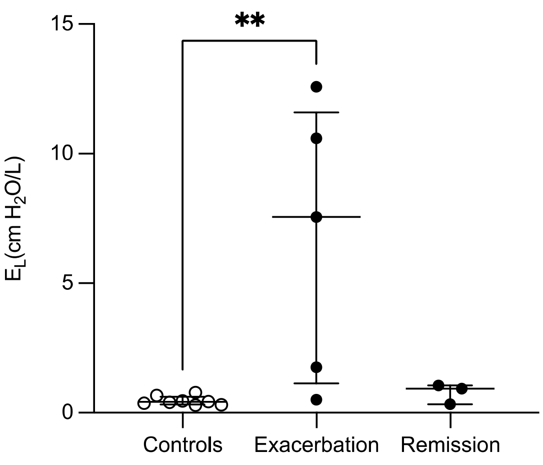

Fig S2. Bronchoalveolar lavage fluid (BALF). Neutrophil percentages in horses with asthma (black dots) in exacerbation (n = 5), in remission (n = 3), and controls (white dots, n = 8) before euthanasia. Horizontal lines represent the median, and the error bars represent the interquartile range. Horses in remission were excluded from BALF analysis.

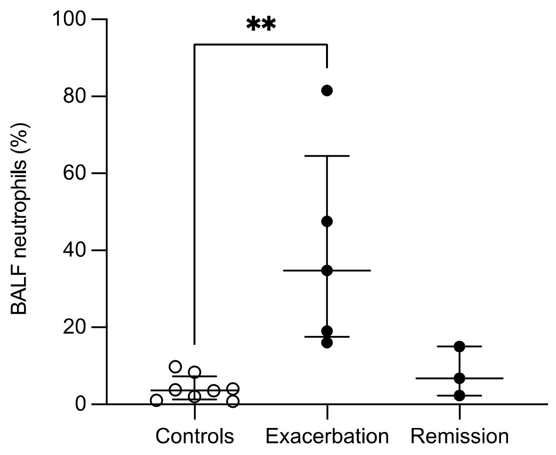

Fig S3. Correlation between histomorphometric measurements. Correlation of smooth muscle area measured by morphometric tracing (ASM-MT) with smooth muscle area measured by point counting analysis (ASM-PC).

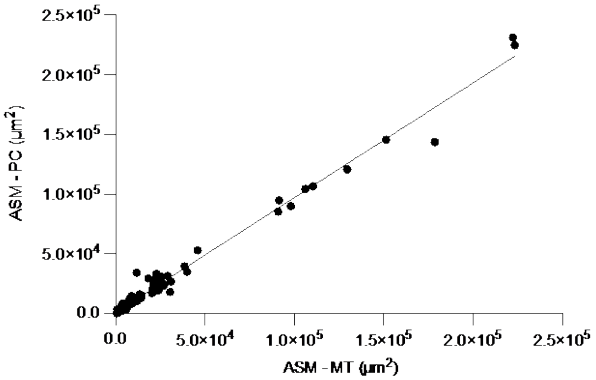

Fig S4. Arterial innervation and arterial smooth muscle area. A) Number of vascular (arterial) nerves (NVN) corrected for internal perimeter squared (μm^2) in horses with asthma (n = 8) and controls (n = 8). Vascular smooth muscle area (VSM) corrected for internal perimeter squared (μm^2) in horses with asthma (n = 8) and controls (n = 8). Horizontal lines represent the median, and the error bars represent the interquartile range.

A

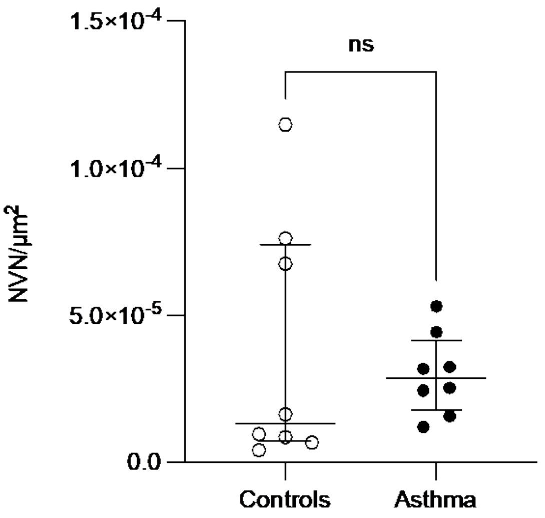

B

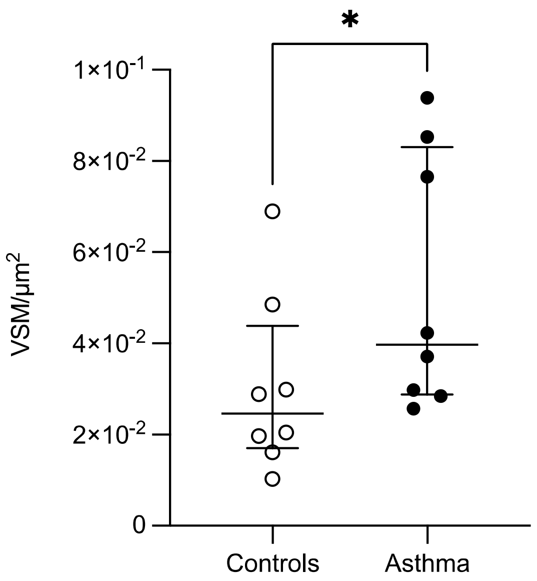

Supplement: Supplementary file 1 — Data S1. Supporting Information Figures. [file JVIM-38-485-s001.pdf]
